# Has the agricultural cooperatives served each member fairly? A new perspective based on utilization level of member services

**DOI:** 10.1371/journal.pone.0294439

**Published:** 2024-01-31

**Authors:** Guoqiang Liu, Chaoyue Chen, Xinhong Fu, Yuying Liu, Nawab Khan, Lei Luo

**Affiliations:** 1 College of Business Administration, Sichuan Vocational College of Finance and Economics, Chengdu, Sichuan, China; 2 College of Management, Sichuan Agricultural University, Chengdu, Sichuan, China; 3 Faculty of Agriculture and Forestry, University of Helsinki, Helsinki, Finland; Xi’an University of Architecture and Technology, CHINA

## Abstract

With the rapid increase of the number of agricultural cooperatives in China, the problem of fake cooperatives has become more and more serious. The core problem is that some members do not use cooperative services, and elite capture phenomenon appears in the organization. Since services are one of the most important public goods attributes of cooperatives, it is important to ensure that more members use them. What are the factors that affect members’ utilization level of cooperative services? Existing research does not provide a comprehensive answer. Based on the micro-survey data of 74 citrus cooperatives and 524 citrus members in China, the article found out that 50.9% of the members did not use any services provided by cooperatives, and only 20.04% of the members used cooperatives’ sales services. So, this study empirically analyzes the factors that influence the use of cooperatives’ services by puns model. The results show that quality of service, service convenience and mountain terrain promote the use of cooperative sales services for members. In addition, cooperative knowledge, planting area, surplus distribution, quality of service, and service convenience significantly increased the utilization leve of cooperative sales services by members. Finally, the study puts forward some suggestions, such as propagating cooperative sales service, improving the quality of cooperative sales service, perfecting cooperative distribution system.

## 1. Introduction

Agricultural cooperatives have the advantages of helping farmers decrease agricultural risks, reduce transaction costs, solve information asymmetry, and improve market negotiation ability, etc. [1–3], and they are regarded as an effective carrier for small farmers to connect with the big market [4–6]. Therefore, countries all over the world generally pay attention to the development of cooperatives. For example, cooperatives account for about half of the European agricultural trade [7, 8]. As for China, almost the Central No. 1 Document will focus on cooperatives every years, and President Xi Jinping also encouraged all parts of the country to develop cooperatives according to local conditions and explored more ways to develop professional cooperatives. With the joint efforts of all sectors of society, Chinese cooperatives have achieved rapid growth since the promulgation of the Law of People’s Republic of China on Farmers’ Professional Cooperatives (LPRCFPC). According to the official data of the Minister of Agriculture and Rural Affairs, more than 2259000 cooperatives had been registered by LPRCFPC at the end of April 2021, about 86 times that of 2007, helping nearly half of the country’s farmers, and with an average of more than 3 cooperatives in each village. At the same time, the service types of cooperatives are increasingly diversified, gradually providing farmers with agricultural sales, materials, capital, technology, information, and other services [9–12].

However, with the number of cooperatives in China booming, the quality of their development has been questioned, such as the falsification of cooperatives [13], cooperative alienation [14], empty-shelled cooperatives [15], and involution of cooperatives [16], which have aroused widespread concern about the development of Chinese cooperatives in all sectors of society. The root cause of these undesirable problems is that many members of cooperatives do not use the services of cooperatives, and the phenomenon of elite prisoners has emerged in the organizations, what was originally a mutually beneficial organization has become a model in which a small number of people benefit [14]. To solve these problems, the Chinese government has published a series of policies, such as the Implementation Plan for Jointly Promoting the Quality Improvement of Farmers’ Professional Cooperatives, the Action for Improving the Quality of Farmers’ Professional Cooperatives, and the Special Cull Work Plan for False Cooperatives of Farmers’ Professional Cooperatives. These documents all address the need to promote access to cooperative services by all members and to promote the overall quality development of cooperatives. However, existing studies have not systematically identified the factors that affect members’ utilization level of cooperative services. So, it is an urgent practical problem to find out the factors that affect members’ utilization of cooperative services, and improve the quality of all-round development of cooperatives.

Service is the essential attributes of cooperatives [17, 18], the key to the service function of cooperatives lies in the active utilization of their services by members [19–21]. Therefore, analyzing the factors influencing members’ utilization of cooperatives’ services is of great significance to perfect the service function of cooperatives, moreover, improve the quality and efficiency of cooperatives. As for the research on the utilization of cooperatives’ services by members, many scholars have directly investigated the behavior of farmers joining the cooperatives. For example, Fischer and Qaim [4] analyzed the determinants of Kenyan banana farmers joining cooperatives. Kumar [22] and Jitmun [23] analyzed the influencing factors of dairy farmers becoming cooperatives members. Ma and Abdulai [24] pointed out that labor force, planting area, computer, and neighbor membership significantly promoted apple farmers in China to join cooperatives. The research of Jia showed that education [25], labor force, non-agricultural experience, village cadres, and small and medium scaled were conducive to guiding pig farmers to participate in cooperatives, while breeding experience significantly inhibited farmers from taking part in cooperatives. Lin et al. [26] found that gender, health, education, labor force, land area, etc. had a significant impact on the participation in cooperative rice farmers. However, these scholars do not pay much attention to the actual utilization condition of cooperatives’ services by farmers after they joined the cooperatives and became members. It seems that the every cooperatives’ members all use services by default. Obviously, this kind of research vaguely examines the members’ utilization of cooperatives’ services.

In fact, members do not necessarily use cooperatives’ services [27, 28]. Sales service is one of the most important service functions of cooperatives [29], but few scholars have investigated the utilization of cooperatives’ sales service by members. Pascucci pointed out that the participation of farmers in cooperatives did not necessarily lead them to sell all their products to cooperatives [30]. Mujawamariya found that there was a phenomenon of double side-selling among members, that is, they used both cooperatives and other channels for sales [31]. The statistical analysis results of Hao et al. [6] showed that 44.0% of members’ apples were sold to wholesalers, 38.1% to small dealers and only 14.4% used cooperatives to sell. The empirical analysis results of Fulton revealed that the surplus capacity of cooperatives [32], cooperatives’ service capacity, and the proportion of wheat income to total household income significantly affected the utilization of cooperatives’ sales service. Cechin thought that the market mechanism [33], bureaucratic mechanism, community mechanism, and democratic mechanism were more perfect, and members were more inclined to use cooperatives’ sales service. Fisher and Qaim further found that the individual characteristics of members, family business characteristics [34], and cooperative characteristics had an impact on whether members use cooperatives’ sales service and the utilization intensity of members.

As for the research on the utilization of cooperative’ services by members, most scholars have directly examined farmers’ behaviors of joining cooperatives and empirically analyzed their influencing factors. However, such studies do not know the specific situation of members’ utilization of cooperatives’ services, that is, to vaguely analyze the members’ utilization of cooperatives’ services. In addition, some scholars seem to acquiesce that the members who join the cooperative all use the cooperative’s services. This is inconsistent with reality. Sales service is one of the most important service functions of cooperatives, few scholars have paid attention to the differences in the utilization of cooperatives’ sales service by members. To sum up, there are few studies on the utilization degree of cooperatives’ sales service by members. In recent years, there has been a growing global demand for Cash crop fruit, such as citrus [35, 36]. Cash crop cultivation, including the commercial fruit, palm oil, rubber and tea, has become an expanding global phenomenon, especially in China and tropical countries [37–39]. Fruit Cash crop, such as oranges, can increase social welfare, particularly raising farmers’ incomes [40, 41]. At present, cash crop cultivation is regarded as a source of export and a critical contributor to economic growth for the producer regions, and has accelerated local economies integration into global economies. More and more researchers focus on the cash crop [42–44]. Therefore, taking China citrus cooperative as an example, this study deeply analyzes the factors affecting the utilization level of cooperatives’ sales service by members, which is innovative to a certain extent.

The remaining structure of this study is arranged as follows: The second part is the theoretical analysis. The third part is the data, variables, and methods. The fourth part is the results. The fifth part is the discussion, and the last part is the conclusions and implications.

## 2. Theoretical analysis

### 2.1. Definition of basic concepts

Members’ utilization level of cooperatives’ sales service can be analyzed from two dimensions. On the one side, we can directly investigate whether members use cooperatives’ sales services or not. on the other side, there are differences in the utilization degree among members using cooperatives’ sales services. Learning from the research of relevant scholars [45, 46], this study uses utilization level to investigate the degree to which members use cooperatives’ sales service. Specifically, utilization level refers to the proportion of citrus output sold by members using cooperatives. Moreover, some scholars use utilization intensity to describe the degree of utilization [47–49]. However, most of the utilization intensity is a quantitative value, while the utilization level is a proportional value, which can better describe the utilization degree of cooperatives’ sales service by members.

### 2.2. Analysis framework

Under the background that citrus production faces multiple risks such as nature, market, policy, and COVID-19, members usually take both risk aversion and maximizing their income into account to make behavioral decisions based on the risk aversion theory and the rational economic man theory [50, 51]. Specifically, if the utilization of cooperatives’ sales service can effectively avoid market risks and the potential income is greater than that brought by non-utilization, members will choose to use cooperatives’ sales service. On the contrary, the members will choose not to use cooperatives’ sales service. Furthermore, this study discusses the factors influencing members’ utilization level of cooperatives’ sales service from two aspects of demand and supply based on the analysis framework of Wu and Ding [10].

From the perspective of the demand side, the individual characteristics of members [52–54] and the characteristics of household management [55, 56] will affect members’ potential demand for cooperatives’ sales service, and then affect the utilization behavior of members. In general, the older the members are, the more vulnerable they are to the influence of the traditional model that production and sales are all completed by themselves, so they are less likely to use cooperatives for sales [57]. Education can improve members’ understanding of cooperatives’ services functions [58, 59], which is conducive to guiding members to use cooperatives’ sales service. The higher the knowledge of cooperatives, the better the members understand the advantages and characteristics of cooperatives’ services, which is good for improving the utilization level of members [60]. Due to the existence of the neighborhood effect, if the neighbor is a member, the member is more likely to use the cooperatives’ sales service [24]. Citrus is a labor-intensive industry, which requires a lot of labor force. Therefore, members with small number of labor force, they have a greater demand for agricultural services [61], so they are more likely to use cooperatives to sell citrus. The larger the citrus’ planting area, the greater the sales risk and pressure faced. To solve these difficulties, members may make deeper use of cooperatives’ sales service [62]. The longer the planting of fruit years, the more likely members are to understand that collective action can obtain relatively high profits [23], so it is more likely to sell citrus by cooperatives. The higher the degree of specialization in agricultural production, the more the family economy of members is dominated by agriculture, so more channels are needed to expand their sales channels, which may increase the utilization level of cooperatives’ sales service [63].

**Fig 1:**
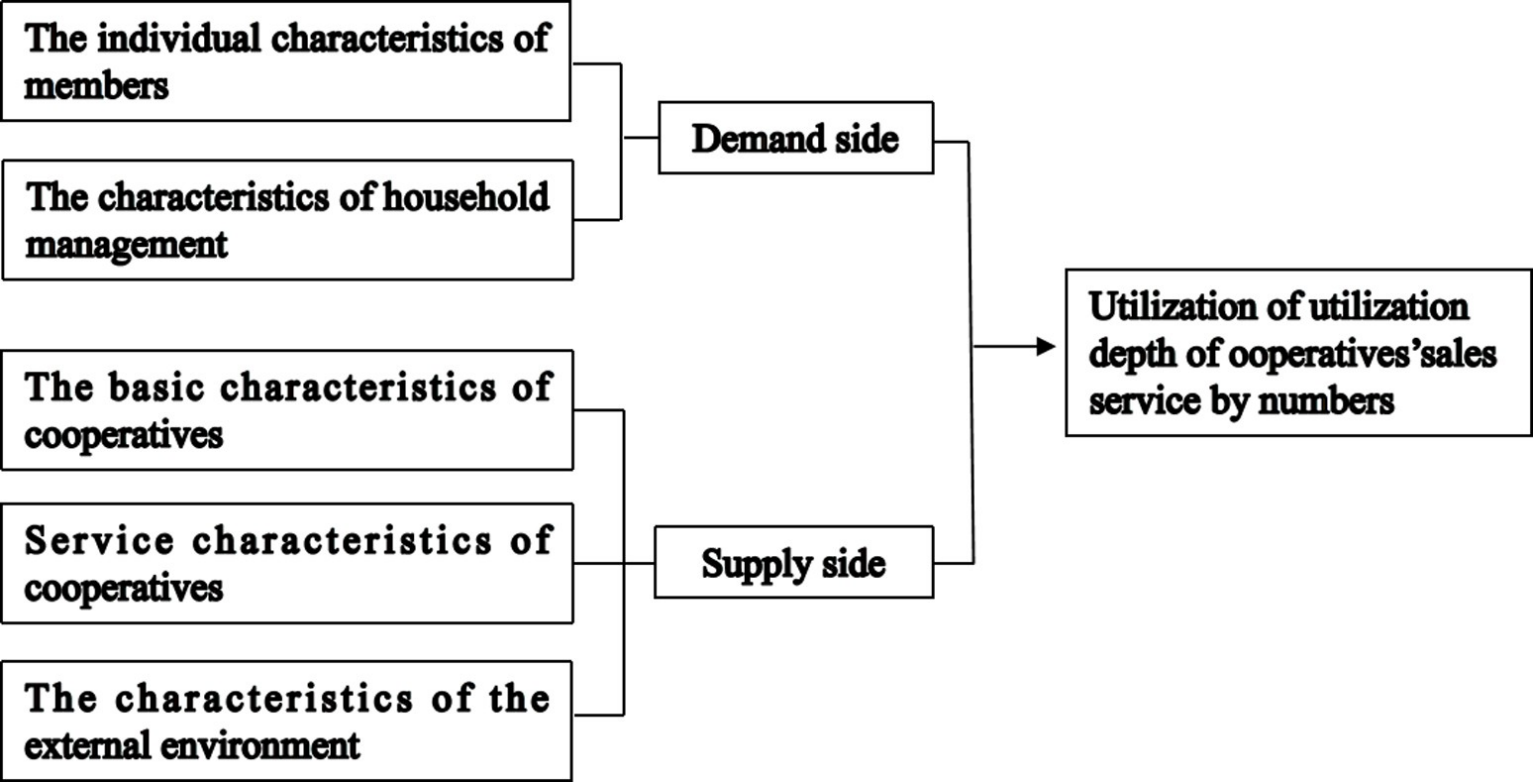
Analysis frame of the utilization of cooperatives’ sales service by members.

From the perspective of the supply side, the basic characteristics of cooperatives will certainly affect the supply of their sales service. Compared with non-demonstration cooperatives, demonstration cooperatives can better provide members with services such as product acquisition, product sales, and business training, which is beneficial for members to use cooperatives’ services [64]. The more perfect the cooperatives’ governance mechanism is, the better the service satisfaction of members will be [65], so the more feasible it is for members to use the cooperatives’ sales service. In addition, the core of cooperatives’ governance mechanism is the decision-making mechanism and the distribution system [1, 3]. The quality of cooperatives’ services also affects the behavior of members [66]. Taking sales service as an example, members are concerned about whether the purchase by cooperatives has a price advantage [67]. Moreover, whether the sales service provided by cooperatives is timely, etc. [68]. The service supply of cooperatives is also affected by the characteristics of the external environment. The cooperative is one of the subjects in the entire agricultural socialization service system, and other service subjects have a certain substitution or complementary effect on cooperatives [69], so the development degree of the external service market has a certain impact on the service utilization of members. Furthermore, the development level of cooperatives in different economic zones is different, and there are differences in their ability to provide services [70], therefore, the utilization level of members may vary (Fig 1).

## 3. Data, variables and methods

### 3.1. Data

Sichuan province is the most widely distributed area of citrus in China. The data used in this study comes from the author’s micro survey of citrus cooperatives and their members in Sichuan province. The survey adopts the combination of typical sampling and random sampling: specifically, in the selection of survey areas, the method of typical sampling is adopted. Due to the lack of statistical data specifically for citrus cooperatives, to ensure enough samples of citrus cooperatives, citrus counties are mainly considered in the selection of sample areas. According to China’s Ministry of Agriculture in Sichuan Province, the author calculated the average citrus yield of each citrus county in Sichuan province in 2018 and 2019 and then obtains the ranking of the top ten citrus countie, namely Anyue, Renshou, Pujiang, Zizhong, Jintang, Yanjiang, Jiang’an, Dongpo, Rong and Danling. Statistics from the Sichuan Provincial Department of Agriculture and Rural Affairs showed that 130 counties (cities and districts) in Sichuan Province produced citrus in 2019, with a total output of 4522264 tons, while the total output of the top 10 citrus counties was 2367069 tons, accounting for 52.34%. Therefore, it is typical to use the top 10 citrus counties to represent the overall situation of citrus in Sichuan.

The investigation is divided into pre-investigation and formal investigation. The pre-investigation was carried out in Jintang County on July 15, 2020, which mainly improves the investigation scheme and questionnaire based on the field feedback, and further formed the final formal investigation scheme and questionnaires. The formal survey was conducted in the remaining 9 counties (districts) from July 20 to August 1, 2020, mainly using the method of typical sampling: first, the Agriculture and Rural Affairs of the sample county (district) provided the list of citrus cooperatives; Secondly, the research team randomly selected 5–12 citrus cooperatives in each sampled county (district); Thirdly, In order to ensure the quality of the survey questionnaires, the survey team conducted a questionnaire survey on the chairman or other council members of cooperatives. At the same time, 5–15 members were randomly selected in each cooperative, and a questionnaire survey was conducted on the head of household; Finally, a total of 90 cooperatives questionnaires were distributed and 80 were recovered, of which 74 were effective, with an effective rate of 92.50%; 650 members questionnaires were distributed and 580 were recovered, of which 524 were valid, with an effective rate of 90.34%. When we conducted our survey in rural areas, we clearly informed everyone about the content of our survey and the purpose of publishing our academic papers, the interviewees gave their informed consent, and all the interviewees were informed of the interview questions, and informed and obtained consent for data analysis, and signed a survey informed consent form to exempt studies reporting on human participants from ethical review. In addition, the study did not include minors. It is therefore exempt from review and approval by the Ethics Committee. In the supplementary documents, we provided a questionnaire with the participants’ signatures to prove that the participants fully agreed to be interviewed and to publish the data.

### 3.2. Variables

#### 3.2.1. Dependent variable

The dependent variable in this study is the utilization level of cooperatives’ sales service by members, that is, the proportion of citrus output sold by cooperatives in last year. The formula for the dependent variable is: *S*1_*i*_/*s*_*i*_, *S*1_*i*_ is the number of oranges that farmer i sold through the cooperative last year, and *s*_*i*_ is the total number of oranges that farmer i sold last year.

As for sales services (see Table 1), nearly 80% of members did not use cooperatives’ sales service, which effectively verifies the view that members do not necessarily use cooperatives’ sales service [6, 30]. Furthermore, there are differences in the utilization level of cooperatives’ sales service among members. The number of members whose utilization level is greater than 0.25 and less than or equal to 0.5 is the largest, but the proportion is only 8.97%. In addition, the members whose utilization level is greater than 0.5 account for only 8.78%. Obviously, the overall utilization level of cooperatives’ sales service by members is low, which is naturally not conducive to the sustainable development of cooperatives. However, the key to the service function of cooperatives lies in the active utilization of their services by members. Therefore, what factors affect the utilization level of cooperatives’ sales service by members will be empirically tested later. In addition, not Limited to the sale of services, the data of this study showed that in 524 samples of members, 50.9% of the members did not use any common services provided by the cooperative, such as agricultural materials, sales, capital, technology, and information services.

**Table 1:** Descriptive statistics of utilization level of cooperatives’ sales service by members.

Utilization level	Quantity	Proportion
**0**	419	79.96
**Greater than 0 and less than or equal to 0.25**	12	2.29
**Greater than 0.25 and less than or equal to 0.5**	47	8.97
**Greater than 0.5 and less than or equal to 0.75**	35	6.68
**Greater than 0.75 and less than or equal to 1**	11	2.10
**Total**	524	100.00

#### 3.2.2. Independent variables

Based on the previous theoretical analysis, the individual characteristics of members, the characteristics of household management, the basic characteristics of cooperatives, the service characteristics of cooperatives, and the characteristics of the external environment may have a potential impact on the utilization level of cooperatives’ sales service by members. Specifically, the individual characteristics of members are described by age, education level, understanding level of cooperatives, and whether neighbors are members. the characteristics of household management are characterized by the labor force, planting area, planting years, and the proportion of Citrus income. The basic characteristics of cooperatives are represented by the demonstration level (The Chinese government classifies cooperatives according to their size and strength, and the higher the level, the stronger the cooperative), the voting method in the membership meeting, and the surplus distribution method. The service characteristics of cooperatives are represented by quality of service and service convenience, and external environmental characteristics are represented by the development degree of the external service market and geographical area. The variable definitions and descriptive statistics are detailed in Table 2.

**Table 2:** Variable definitions and descriptive statistics.

Variables	Definition	Mean (Standard deviation)
**The dependent variable**		
**Service utilization**	1 = utilization, 0 = non-utilization	0.20 (0.40)
**Utilization level**	the proportion of citrus output sold by cooperatives in last year	0.11 (0.24)
**Independent variable**		
**Age**	Actual value (years)	55.27 (9.93)
**Education level**	Actual years of education (years)	7.44 (3.55)
**Knowledge of cooperatives**	Five categorical variables, 1–5 in ascending order	3.02 (1.14)
**Whether neighbors are members**	1 = yes, 2 = no	0.77 (0.42)
**Labor**	Actual number(person)	2.07 (0.97)
**Planting area**	Actual value (mu)	6.14 (4.82)
**Planting time**	Actual years (years)	13.01 (9.83)
**Proportion of citrus income**	Citrus income divided by annual household income (%)	62.39 (31.23)
**Demonstration level**	Demonstration level of co-operatives: 1 = non-demonstration, 2 = county level, 3 = municipal level, 4 = provincial level, 5 = national level	2.21 (1.31)
**Voting method in the membership meeting**	1 = “one person, one vote”, 0 = other	0.65 (0.48)
**Surplus distribution method**	1 = according to trading volume or shares, 0 = other	0.46 (0.50)
**Quality of service**	Five categorical variables, 1–5 in ascending order	2.44 (0.94)
**Service convenience**	Five categorical variables, 1–5 in ascending order	3.00 (1.15)
**The development degree of external service market**	Five categorical variables, 1–5 in ascending order	3.29 (0.75)
**Geographical area**	1 = Chengdu Plain, 2 = Northeast Sichuan, 3 = South Sichuan	1.59 (0.86)

### 3.3. Methods

The Tobit model is usually used to investigate the degree of an individual’s behavior, but the model regards behavior decision-making and degree decision-making as a process for analysis. However, in practice, behavior decision-making and degree decision-making are two decision-making processes with obvious time order, that is, the individual first decides whether to make a specific behavior and then decides the degree of the specific behavior. To make up for the deficiency of the Tobit model in analyzing such problems, Cragg proposed the Double Hurdle model. At present, the Double Hurdle model has been widely used in the analysis of individual behavior decision-making [71–73].

The Double Hurdle model relaxes the Tobit model assumptions, which is also called the generalized Tobit model. Moreover, it can have different estimation coefficients in the behavior decision-making equation and degree decision-making equation. The Double Hurdle model is suitable for analyzing the influencing factors of individuals in two different decision-making stages of economic behavior, and its essence is the combination of Probit model and Truncated model. The Double Hurdle model is set as follows:

$$\left\{\begin{array}{cc} Z_{i}^{*}=\alpha X_{1i}+\mu_{i} & \mu_{i}-N(0,1)\\ Y_{i}^{*}=\beta X_{2i}+v_{i} & v_{i}-N(0,1)\\ \left\{\begin{array}{c} Z_{i}=1,Z_{i}^{*}>0\\ Z_{i}=1,Z_{i}^{*}\leq 0 \end{array}\right. & \end{array}\right.$$ (1)

$$\left\{\begin{array}{l} Y_{i}=Y_{i}^{*},\;\text{if}\;Z_{i}^{*}>0\;\text{and}\;Z_{i}=1\\ Y_{i}=0,\;\text{if}\;Z_{i}^{*}\leq 0 \end{array}\right.$$ (2)

$$L(\alpha ,\beta ,\sigma )=\prod_{i=1}^{n}{\left[1-\Phi \left(X_{1i}\right)\right]}^{1(\omega -0)}\prod_{i=1}^{n}\Phi \left(X_{1i}\right)\frac{1}{\sqrt{2\pi }\sigma }exp\left[\frac{-{\left(Y-X_{2i}\beta \right)}^{2}}{2\sigma^{2}}\right]/{\left[\Phi \left(X_{2i}\right)\right]}^{1(\omega -1)}$$ (3)

In the above equation, (.) is an indicator function. If the formula in parentheses holds, it is assigned as 1, otherwise, it is assigned as 0, that is, when *Y*_*i*_ is greater than 0, it is assigned as 1, otherwise, it is assigned as 0. α and β represent the coefficients of independent variable *X* in the decision equation and degree equation respectively.

The utilization behavior of cooperatives’ sales service by members can also be regarded as the combination of two decision-making stages. The first stage is whether members make use of cooperatives’ sales service or not, that is, the decision-making model; The second stage determines the utilization level of cooperatives’ sales service, that is, the utilization level model. Therefore, to deeply analyze the utilization behavior of cooperatives’ sales service by members, this study uses Double Hurdle model to empirically test the factors influencing the utilization level of cooperatives’ sales service by members.

## 4. Results

Double Hurdle mode is used in this paper to empirically investigate the factors influencing the utilization level of cooperatives’ sales service by members. Firstly, the multicollinearity between independent variables is tested. From Table 3, the mean value of the variance inflation factor (VIF) of each variable is 1.25 and the maximum VIF is 1.44, indicating that there is no significant multicollinearity in the model. Afterward, this study uses stata15.0 statistical analysis software for model estimation.

**Table 3:** Results of multicollinearity test of independent variables.

Variables	VIF	1/VIF
**Age**	1.42	0.70
**Education level**	1.44	0.70
**Knowledge of cooperatives**	1.28	0.78
**Whether neighbors are members**	1.11	0.90
**Labor**	1.09	0.92
**Planting area**	1.38	0.73
**Planting time**	1.35	0.74
**Proportion of citrus income**	1.34	0.75
**Demonstration level**	1.19	0.84
**Voting method in the membership meeting**	1.19	0.84
**Surplus distribution method**	1.09	0.91
**Quality of service**	1.11	0.90
**Service convenience**	1.22	0.82
**The development degree of the external service market**	1.11	0.90
**Northeast Sichuan**	1.36	0.73
**Southern Sichuan**	1.36	0.74
**Mean VIF**	1.25	

Table 4 shows the estimated results of the Double Hurdle model. The Log-likelihood is -50.319. The chi-square overall of the model is 343.073, and it is significant at the 1% level, indicating that the overall fitting effect of the Double Hurdle model is significant, which applies to the empirical analysis of this paper. It can be known from the following that the Double Hurdle Model is composed of two hurdles. The first hurdle is the utilization decision-making of cooperatives’ sales service by members, that is, the decision-making model, which corresponds to the left half of Table 4, and the second hurdle is the utilization level decision-making of cooperatives’ sales service by members, that is, the utilization level model, which corresponds to the right half of Table 4. The specific analysis is as follows:

**Table 4:** Estimation results of the Double-Hurdle model.

Types of characteristic	Variables	Decision-making model		Utilization level model	
		Coefficient	Z value	Coefficient	Z value
**Individual characteristics**	Age	0.006	0.590	-0.001	-0.671
	Education level	0.008	0.289	0.004	1.161
	Knowledge of cooperatives	-0.022	-0.268	0.034***	3.303
**Family management characteristics**	Whether neighbors are members	0.683**	2.467	0.005	0.109
	Labor	-0.160	-1.512	0.001	0.083
	Planting area	-0.015	-0.732	0.016***	6.295
	Planting time	-0.008	-0.797	-0.001	-0.536
	Proportion of citrus income	0.002	0.638	0.000	0.299
**Basic characteristics of cooperatives**	Demonstration level	-0.015	-0.213	0.012	1.301
	Voting method in the membership meeting	0.004	0.020	0.027	1.103
	Surplus distribution method	-0.141	-0.788	0.051**	2.358
**Service characteristics of cooperatives**	Quality of service	0.859***	7.417	0.029**	2.188
	Service convenience	0.711***	7.425	0.051***	3.478
**External environment characteristics**	The development degree of the external service market	-0.101	-0.864	-0.005	-0.311
	Northeast Sichuan	0.634**	2.153	-0.035	-0.995
	Southern Sichuan	0.199	0.906	-0.026	-0.945
	Constant	-5.800	-5.916	0.003	0.021
	Total sample	524			
	Log likelihood	-50.319			
	Chi-square overall	343.073^***^			

Note: *, ** and *** are significant at 10%, 5% and 1% levels respectively

### 4.1. Influence of the individual characteristics of members

The knowledge of cooperatives passes the significance test at the 1% level in the utilization level model, and the coefficient is positive, indicating that the higher the knowledge of cooperatives, the higher the utilization level of cooperatives’ sales service by members, which is consistent with the research conclusion of Ito et al. [60]. Because the more members know about cooperatives, the more members can recognize the advantages of cooperatives’ sales service and help themselves make reasonable economic choices. Thus, when guiding members to use cooperatives’ sales service, we should pay attention to the publicity of cooperatives and enhance members’ understanding of cooperatives.

Whether neighbors are members passes the significance test at the 1% level in the decision-making model, and the coefficient is positive, indicating that members whose neighbors are members are more likely to use cooperatives for sales, which is in line with the research conclusion of Ma and Abdulai [24]. Chinese rural society has the typical characteristics of an acquaintance society and semi-acquaintance society, and the influence between relatives and friends is strong. Hence, individual behavior is potentially affected by neighborhood or group behavior. However, whether neighbors are members does not pass the significance test at the 10% level in the utilization level model.

### 4.2. Influence of the characteristics of household management

The planting area passes the significance test at the 1% level in the utilization level model, and the coefficient is positive, indicating that the larger the planting area is, the higher the utilization level of cooperatives’ sales service by members, which response to the research of Ma et al. [62]. Because the larger the planting area, the greater the risks faced by members, such as extreme weather, market price fluctuations, COVID-19, etc. Hence, members prefer relatively stable sales channels, such as cooperatives.

### 4.3. Influence of the basic characteristics of cooperatives

The surplus distribution method passes the significance test at the 5% level in the utilization level model, and the coefficient is positive, showing that the members whose surplus is distributed according to the trading volume or share may have a higher utilization level of cooperatives’ sales service, which response to the research conclusion of Bijman et al. [74]. Because if the cooperative distributes surplus according to trading volume or shares, the potential income of members is higher. As a rational person, the member will naturally increase the proportion of sales using cooperatives [51]. Therefore, the distribution system has an important impact on the utilization level of cooperatives’ sales service by members.

### 4.4. Influence of service characteristics of cooperatives

The quality of service passes the significance test at the level of 1% and 5% respectively in the decision-making model and utilization level model, and the coefficients are all positive, which indicates that the higher the quality of service, the higher the possibility of members using cooperatives to sell services, and the higher the proportion of citrus sold by members using cooperatives. Because the quality of service is a key indicator to measure the sales service quality of cooperatives, which is conducive to improving the welfare of members [67]. Therefore, the higher the quality of service, members naturally tend to use cooperatives for sales and this will increase the sales proportion by cooperatives.

The service convenience passes the significance test at the 1% level in the decision-making model and the utilization level model, and the coefficients are all positive, indicating that the higher the service convenience, the higher the possibility of members using cooperatives to sell, and the higher the proportion of citrus sold by members using cooperatives. Because the more convenient the service is, the more convenient the members can make use of cooperatives’ sales service, which is conducive to reducing members’ transaction costs and then indirectly increasing members’ welfare [68].

### 4.5. Influence of the characteristics of external environment

Only Northeast Sichuan passes the significance test at the 5% level in the decision-making model, and the coefficient is positive, indicating that compared with the members from Chengdu Plain Economic Zone, members coming from Northeast Sichuan are more likely to use cooperatives for sales, which partly responds to the research of Wadsworth [29] and Sebhatu et al. [70]. The northeast of Sichuan province is a typical mountainous region, and there are many problems in the sale of agricultural products, such as transportation difficulties and high transaction costs. Obviously, we should pay attention to the regional differences in the service supply of cooperatives. We need to further summarize and refine the experience of cooperatives’ sales service in mountainous region, and actively do a good job in publicity and promotion.

### 4.6. Robustness analysis

To verify the robustness of the model and samples, considering that members over 70 years old are greatly affected by the traditional model of self-production and self-marketing, age may have a potential impact on the utilization level of cooperatives’ sales service by members. Therefore, to test the robustness of the model results above, this study only uses the member samples under the age of 70 for the estimation with the Double Hurdle model, and the detailed results are shown in Table 5.

**Table 5:** Robustness analysis results.

Variables	Decision-making model		Utilization level model	
	Coefficient	Z value	Coefficient	Z value
**Age**	0.005	0.425	0.000	-0.076
**Education level**	0.010	0.327	0.006	1.581
**Knowledge of cooperatives**	-0.030	-0.341	0.027^**^	2.340
**Whether neighbors are members**	0.642^**^	2.159	0.009	0.204
**Labor**	-0.172	-1.574	0.003	0.253
**Planting area**	-0.021	-0.944	0.015^**^	5.981
**Planting time**	-0.007	-0.613	0.000	-0.230
**Proportion of citrus income**	0.001	0.406	0.000	0.565
**Demonstration level**	0.013	0.177	0.012	1.355
**Voting method in the membership meeting**	-0.030	-0.154	0.021	0.874
**Surplus distribution method**	-0.242	-1.283	0.045^**^	2.062
**Quality of service**	0.855^***^	6.979	0.025^*^	1.708
**Service convenience**	0.725^***^	7.026	0.056^***^	3.581
**The development degree of the external service market**	-0.066	-0.545	0.002	0.109
**Northeast Sichuan**	0.674^**^	2.068	-0.015	-0.394
**Southern Sichuan**	0.205	0.905	-0.027	-0.992
**Constant**	-5.772	-5.391	-0.068	-0.491
**Total sample**	485			
**Log-likelihood**	-44.613			
**Chi-square overall**	302.645^***^			

Note: *, ** and *** are significant at 10%, 5% and 1% levels respectively

The robustness test results show that the Log-likelihood is -44.613. The chi-square overall of the model is 302.645, and it is significant at the 1% level, indicating that the overall fitting effect of the double hurdle model is significant. Comparing Tables 4 and 5, there is little difference in the coefficient size and significance of each independent variable, indicating that the analysis results of the main model are relatively robust.

## 5. Discussion

Cooperative is a spontaneous union of farmers, who have the right to decide whether to use the services of the cooperative, and members joining cooperatives do not necessarily use cooperative services. The services provided by cooperatives mainly include agricultural materials, sales, capital, technology, information, etc. According to the survey data, 50.9% of the members did not use any services provided by cooperatives. As for sales services, only 20.04% of the members used cooperatives’ sales services. Obviously, the utilization of cooperatives’ services by members is poor. However, in the existing research on the utilization of cooperatives’ services by members, most scholars have directly investigated the influencing factors of farmers’ participation in cooperatives [22, 24, 26]. It seems that members will use cooperatives’ services by default, but this is not the true in China. Therefore, when evaluating the development of cooperatives, we should not only pay attention to the number of members in cooperatives but also pay attention to the number of members who use cooperatives’ services.

The utilization level of cooperatives’ services by members is comprehensively affected by the two dimensions of demand and supply. The active utilization of cooperatives’ services by members can promote the sustainable development of cooperatives [19, 21]. Although some researchers empirically investigate the factors influencing members’ service utilization from the aspects of the individual characteristics of members. Family management characteristics, cooperative characteristics, and the characteristics of the external environment [32, 33], few scholars pay attention to the differences in the utilization of cooperatives’ services by members [6, 31]. At the same time, there are few empirical studies on the factors influencing the utilization level of cooperatives’ services by members. Therefore, this study empirically analyzes the factors influencing the utilization level of cooperatives’ services by members from five aspects: the individual characteristics of members, the characteristics of household management, the basic characteristics of cooperatives, the service characteristics of cooperatives, and the characteristics of the external environment. Specifically, the first two aspects are considered from the perspective of members’ service demand, while the last three aspects are considered from the perspective of service supply. Furthermore, Like Part 4 for detail, the relevant conclusions of this study effectively echo the previous studies.

Service is the essential attributes of cooperatives [17]. Enabling each member to access the services of the cooperative is fundamental to the overall development of the organization. From the demand and supply of cooperative services, on the one hand, we should pay attention to the supply port of the service, and the key to improve the utilization level of the member’s service is to improve the service of the organization, we should objectively look at the current difficulties faced by cooperative development, and strive to improve the quality of cooperative services. Take the sales service as an example, the emphasis should be put on improving the service quality and service convenience of the cooperative. In addition, there should be a step-by-step approach, with the current focus on improving the distribution system and promoting surplus distribution by volume of transactions or shares, so as to facilitate the formation of a close community of interests between cooperatives and their members, and then improve the utilization level of cooperative services. On the other hand, attention should be paid to the farmer’s demand port of the service, the main body of the service utilization is the cooperatives members, and the cooperative knowledge and awareness of the members should be constantly improved through publicity and training. The government should guide all farmers in the radiation region to join the cooperative to form a group demonstration and lead effect, and to promote farmers to use cooperative services as a whole. Members are encouraged to scale up their production and are stimulated to generate more demand for sales services and to take advantage of cooperatives services. In addition, because of the limitation of the mountainous terrain to the traffic, the cooperatives in mountainous terrain should strengthen the marketing service and improve the service utilization level of more farmers

There are still some limitations in this study, and future research can be improved from the following aspects:

(1) COVID-19 broke out in early 2020 and may have some influence on the results. Survey shows COVID-19 mainly affects cooperatives’ sales early, however, this dilemma has been alleviated in time with the help of the government. Moreover, affected by previous sales inertia, there was no significant change in the members using cooperatives’ sales service before and after COVID-19. Further analysis can be done using data from previous years before COVID-19, moreover, compared the results with this study.
(2) Taking citrus cooperatives as an example, this study analyzes the factors influencing the utilization level of cooperatives’ sales service by members. It is worth noting that there are many kinds of cooperatives in other industries in China, such as rice cooperatives, apple cooperatives, pear cooperatives, etc. Therefore, it can be studied by taking these cooperatives as examples to check whether the conclusions of this study are consistent with them.
(3) This study takes citrus cooperatives in Sichuan province as an example. Due to China’s vast territory and abundant resources, the situation of citrus cooperatives in Sichuan cannot represent the whole country. Therefore, we can take the citrus cooperatives in other provinces as an example to conduct an empirical study, and furthermore, to investigate whether the conclusions of this study are applicable to other provinces.

## 6. Conclusions and implications

Based on the micro survey data of 74 citrus cooperatives and 524 citrus cooperatives members in citrus counties from China, this study empirically analyzes the factors influencing members’ utilization level of cooperatives’ sales service by using the Double Hurdle model. The main conclusions are as follows:

(1) 50.9% of the members did not use any services provided by cooperatives, and only 20.04% of the members used cooperatives’ sales services. (2) Whether neighbors are members, quality of service, service convenience, and mountainous terrain significantly promote members’ utilization of cooperatives’ sales service. (3) Cooperatives knowledge, planting area, surplus distribution method, quality of service, and service convenience significantly improve the utilization level of cooperatives’ sales service by members.

Finally, this study puts forward the following policy suggestions:

(1) Vigorously publicize cooperatives’ sales service. Actively publicize the advantages and characteristics of cooperatives’ sales service, which can enhance farmers’ understanding of cooperatives, and further improve the utilization level of cooperatives’ sales service by members. (2) Promote the quality and efficiency of cooperatives’ sales service. In the context of citrus sales facing various risks such as nature, market, policy, and COVID-19, cooperatives should focus on improving their operateability to increase the welfare of members. At the same time, cooperatives are supposed to improve their service convenience to reduce the transaction cost of members’ citrus sales, which enhances members’ sense of access to the cooperatives’ sales service. (3) Improve the distribution system of cooperatives. In the process of standardized construction of cooperatives, the distribution system is the key to the democratic management of cooperatives. We should actively promote the way of surplus distribution according to the trading volume or shares, protect the rights and interests of members, and then promote the utilization of cooperatives’ sales service by members. (4) Continuous improvement of members’ knowledge and awareness of cooperation through advocacy training. We should guide all farmers in the radiation areas to join cooperatives, form a group demonstration and lead effect, and promote farmers to use cooperative services as a whole. Members are encouraged to scale up production and are stimulated to generate greater demand for marketing services and to make use of cooperative services. And the cooperatives in mountainous terrain should strengthen the marketing service and improve the service utilization level of more farmers.

## Supporting information

S1 Data (XLSX)
